# Azithromycin induces anti-viral effects in cultured bronchial epithelial cells from COPD patients

**DOI:** 10.1038/srep28698

**Published:** 2016-06-28

**Authors:** Mandy Menzel, Hamid Akbarshahi, Leif Bjermer, Lena Uller

**Affiliations:** 1 Respiratory Immunopharmacology, Department of Experimental Medical Science, Lund University, Sweden; 2 Lung medicine and Allergology, Department of Clinical Sciences, Lund University, Sweden

## Abstract

Rhinovirus infection is a major cause of chronic obstructive pulmonary disease (COPD) exacerbations and may contribute to the development into severe stages of COPD. The macrolide antibiotic azithromycin may exert anti-viral actions and has been reported to reduce exacerbations in COPD. However, little is known about its anti-viral actions on bronchial epithelial cells at clinically relevant concentrations. Primary bronchial epithelial cells from COPD donors and healthy individuals were treated continuously with azithromycin starting 24 h before infection with rhinovirus RV16. Expression of interferons, RIG-I like helicases, pro-inflammatory cytokines and viral load were analysed. Azithromycin transiently increased expression of IFNβ and IFNλ1 and RIG-I like helicases in un-infected COPD cells. Further, azithromycin augmented RV16-induced expression of interferons and RIG-I like helicases in COPD cells but not in healthy epithelial cells. Azithromycin also decreased viral load. However, it only modestly altered RV16-induced pro-inflammatory cytokine expression. Adding budesonide did not reduce interferon-inducing effects of azithromycin. Possibly by inducing expression of RIG-I like helicases, azithromycin increased rhinovirus-induced expression of interferons in COPD but not in healthy bronchial epithelium. These effects would reduce bronchial viral load, supporting azithromycin’s emerging role in prevention of exacerbations of COPD.

Chronic obstructive pulmonary disease (COPD) is a heterogeneous disease, characterized by irreversible airflow obstruction and an exaggerated chronic inflammation of the airways. COPD affects about 64 million people worldwide and is estimated to become the third leading cause of death by 2030 (WHO 2013).

COPD is known as a progressive disease. There is a strong association between decline in FEV_1_ and the number of exacerbations patients experience^1^,^2^. Hence severity appears to be determined in part by the frequency of exacerbations. Infections with respiratory viruses cause 20–55% of all COPD exacerbations, with rhinovirus being the most prominent virus detected^3^,^4^. While only a small percentage develop severe disease, they account for up to 75% of all healthcare cost spending for COPD^5^.

Current medications like inhaled corticosteroids do not effectively reduce airway inflammation that is most intense during exacerbation in COPD^6^. Hence, better treatment options are needed, especially for patients with severe disease.

The term “macrolide” joins a group of anti-bacterial agents, composed of a 12- to 16-atom large lactone ring. Their anti-bacterial action comprises of interfering with bacterial protein synthesis by binding to the 50S ribosomal subunit^7^. Recent studies have shown that macrolide antibiotics also display anti-inflammatory and anti-viral activities by variably affecting cytokine expression^8^,^9^,^10^ and reducing susceptibility to viral infections^11^,^12^. In animal models selected macrolides have been found to suppress recruitment and influx of neutrophils after stimulation with lipopolysaccharide^13^,^14^. These compounds have also exhibited anti-inflammatory actions, involving variable inhibition of cytokine release, in different kinds of inflammatory disease models^15^,^16^,^17^.

The first macrolide antibiotic discovered was erythromycin, consisting of a 14-membered lactone ring. Due to erythromycin’s short half-life and adverse effects macrolides with improved features were developed. Azithromycin has a 15-membered ring containing a nitrogen, which gives it an extended serum half-life, improved bioavailability and a greater acid stability over 14-membered macrolides like erythromycin, roxithromycin and clarithromycin^18^,^19^. Together with its pharmacological profile of action this makes azithromycin a suitable candidate for drug intervention in respiratory diseases. Clinical trials using the macrolide antibiotic azithromycin for intervention report a reduction in exacerbation frequency together with an improvement of quality of life in COPD patients^20^,^21^.

Clinical studies aimed at prevention of COPD exacerbations have generally used 250–500 mg azithromycin daily. This dose translates to a concentration of azithromycin less than 1 μM in blood plasma and bronchial washings. Despite azithromycin’s large volume of uptake into tissues only concentrations of 10 μM are achieved in the lung. Even when pushing the dose of azithromycin to 1000 mg, lung tissue levels are below 25 μM^22^. Accordingly, in cell culture studies azithromycin should ideally produce effects at 10 μM or less. By producing anti-viral proteins such as interferons the airway epithelium can mount a defence against invading respiratory viruses. Recent *in vitro* studies reported that azithromycin augments rhinovirus-induced interferon expression in bronchial epithelial cells from healthy donors and children with cystic fibrosis^23^,^24^. Although interesting, in these studies 50 μM azithromycin was required to produce significant anti-viral effects.

Patient-derived airway epithelial cells may well maintain their impaired innate immune response in culture^25^. As an example, we have previously shown that primary bronchial epithelial cells from COPD patients may over-produce a central cytokine such as thymic stromal lymphopoietin (TSLP) in response to viral stimulation^26^,^27^. For this study we hypothesized that rhinovirus-infected bronchial epithelium from COPD donors may respond to azithromycin differently than healthy epithelial cells with regard to any inducement of interferon expression. In view of clinical reports of effect on exacerbations we have focused on the possibility that anti-viral effects may be produced by clinically relevant concentrations of azithromycin in bronchial epithelial cells obtained from COPD donors.

We demonstrate here that clinically relevant concentrations of azithromycin induced transient expression of type I and III interferons in un-infected cells. Furthermore, azithromycin produced marked and sustained augmentation of rhinovirus infection-induced interferon responses and expression of RIG-I like helicases and it also decreased viral load. The azithromycin-induced interferon expression occurred in primary bronchial epithelial cells from COPD patients but not in cells obtained from healthy controls.

## Results

### Azithromycin augments rhinovirus-induced type I and III interferon expression in bronchial epithelial cells from COPD donors

**Figure 1:**
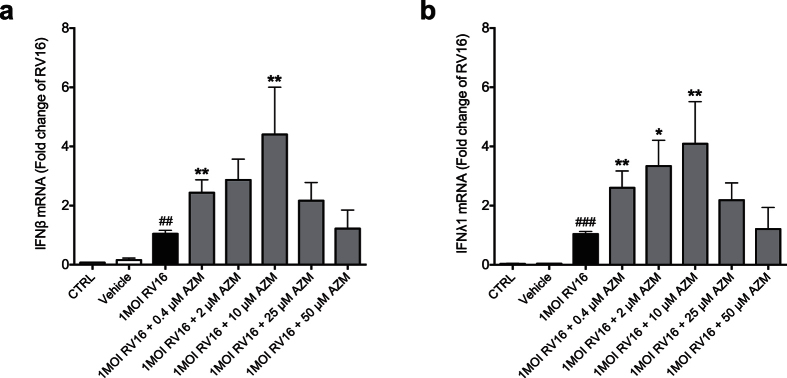
Azithromycin (AZM) augments rhinovirus-induced type I and III interferon expression in bronchial epithelial cells from COPD patients. HBECs from COPD patients were pre-treated with azithromycin for 24 h before infection with 1MOI RV16 and continuous throughout the experiment. Cells were harvested for gene expression analysis 24 h post infection. Gene expression levels of IFNβ (**a**) and IFNλ1 (**b**) were measured by real-time PCR and data is presented as mean ± standard error of the mean (SEM) fold change of RV16 relative to UBC/GAPDH expression. Comparison of different groups was performed by Kruskal-Wallis with Wilcoxon post testing. ^#^p < 0.05, ^##^p < 0.01, ^###^p < 0.001 vs. control; *p < 0.05, **p < 0.01 AZM vs. RV16. Data was obtained from 5 donors.

Infection of bronchial epithelial cells from subjects with COPD with the major group rhinovirus RV16 led to a significant up-regulation of type I interferon (IFNβ) and type III interferon (IFNλ1) gene expression 24 h post infection (Fig. 1). Treatment with azithromycin beginning 24 h prior to infection with RV16 and continuous throughout the experiment further augmented interferon expression (Fig. 1). The effects of azithromycin were concentration-dependent, meaning that a dose of 0.4–10 μM azithromycin in combination with the virus achieved the highest interferon-stimulating effect.

### Azithromycin does not display cytotoxic effects in primary bronchial epithelial cells

To investigate if the induction of interferons by azithromycin was associated with cytotoxic effects of this drug, levels of lactate dehydrogenase (LDH), a crude cell death marker, were measured in cell supernatants. Cells infected with RV16 released LDH, which reflects a successful viral infection. Negligible levels of LDH were obtained after azithromycin treatment alone while treatment with azithromycin in combination with viral infection actually reduced RV-induced LDH levels (Suppl. Fig. S1).

### Azithromycin up-regulates RIG-I like helicases after viral infection

**Figure 2:**
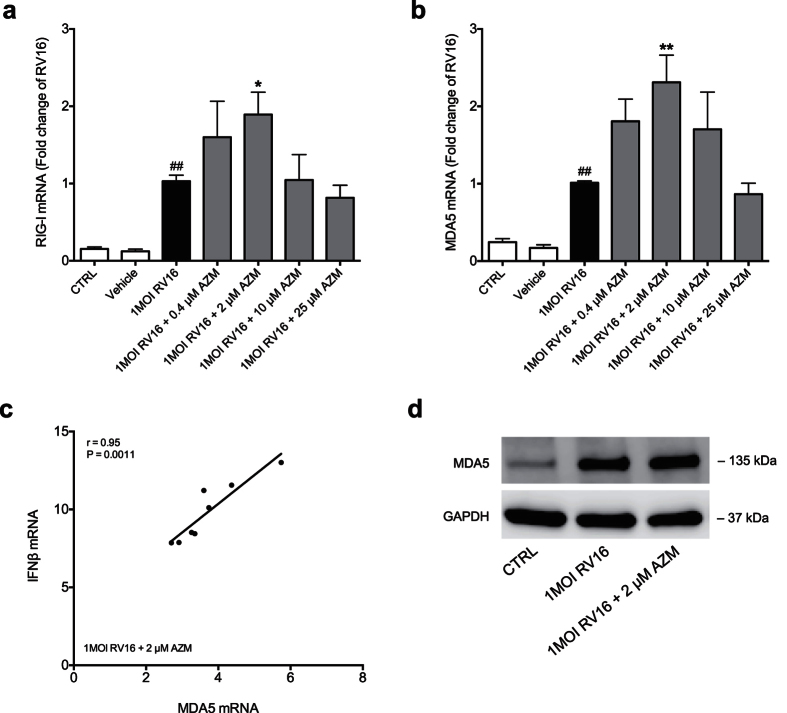
Azithromycin (AZM) up-regulates rhinovirus-induced expression of RIG-I like helicases in bronchial epithelial cells from COPD patients. HBECs from COPD patients were pre-treated with azithromycin for 24 h before infection with 1MOI RV16 and continuous throughout the experiment. Cells were harvested for gene expression analysis 24 h post infection. Gene expression levels of RIG-I (**a**) and MDA5 (**b**) were measured by real-time PCR and data is presented as mean ± standard error of the mean (SEM) fold change of RV16 relative to UBC/GAPDH expression. Comparison of different groups was performed by Kruskal-Wallis with Wilcoxon post testing. ^#^p < 0.05, ^##^p < 0.01 vs. control; *p < 0.05, **p < 0.01 AZM vs. RV16. Data was obtained from 5 donors. Correlation analysis between MDA5 and IFNβ mRNA (**c**) was performed. Correlations were analysed by Spearman. For correlation with a P-value below 0.05, and thus regarded statistically significant, linear regression was employed. A representative Western Blot image of MDA5 protein is shown (**d**).

Next we wanted to see if the up-regulation of interferon expression was associated with an over-expression of the pattern recognition receptors RIG-I and MDA5. Expression of RIG-I like helicases has previously been described to play a role in interferon signalling^26^. Cells were pre-treated with azithromycin before infection with RV16. Viral infection led to a significant induction of RIG-I and MDA5 24 h post infection (Fig. 2a,b). Similar to its effect on interferon expression azithromycin dose-dependently further up-regulated pattern recognition receptor expression (Fig. 2a,b). To further investigate if the induction of interferons and RIG-I like helicases by azithromycin is related we employed correlation analysis for each dose of azithromycin. At 2 μM azithromycin there was a significant correlation between MDA5 and IFNβ (p < 0.01, Fig. 2c) but not between RIG-I and IFNβ (data not shown). Also, 2 μM azithromycin up-regulated rhinovirus-induced expression of MDA5 on protein level (Fig. 2d).

### Azithromycin induces expression of interferons independent of viral infection

**Figure 3:**
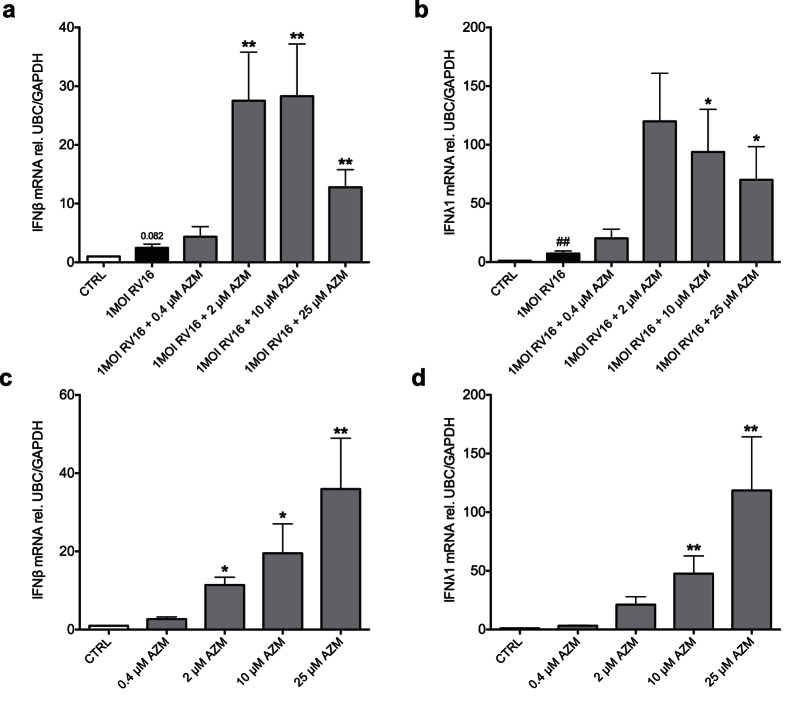
Azithromycin (AZM) induces early type I and III interferon expression independent of rhinoviral infection in bronchial epithelial cells from COPD patients. HBECs from COPD patients were pre-treated with azithromycin for 24 h before infection with 1MOI RV16 and continuous throughout the experiment. Cells were harvested for gene expression analysis 8 h post infection (**a**,**b**). HBECs from COPD patients were pre-treated with azithromycin for 24 h and continuous throughout the experiment. Cells were harvested for gene expression analysis at 8 h (**c**,**d**). Gene expression levels of IFNβ (**a**,**c**) and IFNλ1 (**b**,**d**) were measured by real-time PCR and data is presented as mean ± standard error of the mean (SEM) fold change of control relative to UBC/GAPDH expression. Comparison of different groups was performed by Kruskal-Wallis with Wilcoxon post testing or Dunn’s post testing. ^#^p < 0.05, ^##^p < 0.01 RV16 vs. control; *p < 0.05, **p < 0.01 vs. RV16. Data was obtained from 5 donors.

By analysing effects already 8 h after RV16 infection we revealed that azithromycin produced marked up-regulation of interferon expression also at this point in time when infection alone had not yet produced more than marginal interferon effects (Fig. 3a,b). This observation prompted further studies to determine if this effect was caused by azithromycin itself. Cells treated with azithromycin as above but not infected with RV16 exhibited increased expression of IFNβ (p < 0.01, Fig. 3c) and IFN λ1 (p < 0.01, Fig. 3d) compared to untreated control at 8 h. At this time point also the RIG-I like helicases RIG-I and MDA5 were increased by azithromycin treatment alone both on gene and protein level (Suppl. Fig. S2). However, the interferon-inducing effect of azithromycin alone was not prominent after 24 h treatment; the marginal effect at 24 h also exhibited no dose-dependency (Suppl. Fig. S3).

### Azithromycin modestly reduces gene expression of TNFα but not CXCL8

**Figure 4:**
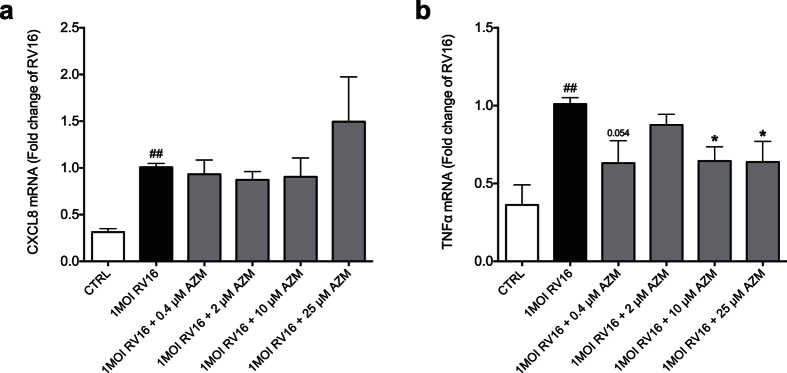
Effects of azithromycin (AZM) on gene expression of pro-inflammatory cytokines in bronchial epithelial cells from COPD patients. HBECs from COPD patients were pre-treated with azithromycin for 24 h before infection with 1MOI RV16 and continuous throughout the experiment. Cells were harvested for gene expression analysis 24 h post infection. Gene expression levels of CXCL8 (**a**) and TNFα (**b**) were measured by real-time PCR and data is presented as mean ± standard error of the mean (SEM) fold change of RV16 relative to UBC/GAPDH expression. Comparison of different groups was performed by Kruskal-Wallis with Wilcoxon post testing. ^#^p < 0.05, ^##^p < 0.01 RV16 vs. control; *p < 0.05 AZM vs. RV16. Data was obtained from 5 donors.

To investigate if the interferon-inducing effect of azithromycin is associated with an over-expression of pro-inflammatory cytokines, we measured gene expression of TNFα and CXCL8. At 24 h post RV16 infection CXCL8 and TNFα expression was significantly elevated by rhinovirus infection compared to control (Fig. 4). While azithromycin tendentially reduced virus-induced TNFα expression (Fig. 4b), it did not alter viral-induced CXCL8 expression (Fig. 4a).

### Azithromycin-induced interferon expression levels in viral-infected bronchial epithelial cells from COPD patients are much higher than in cells from healthy donors

**Figure 5:**
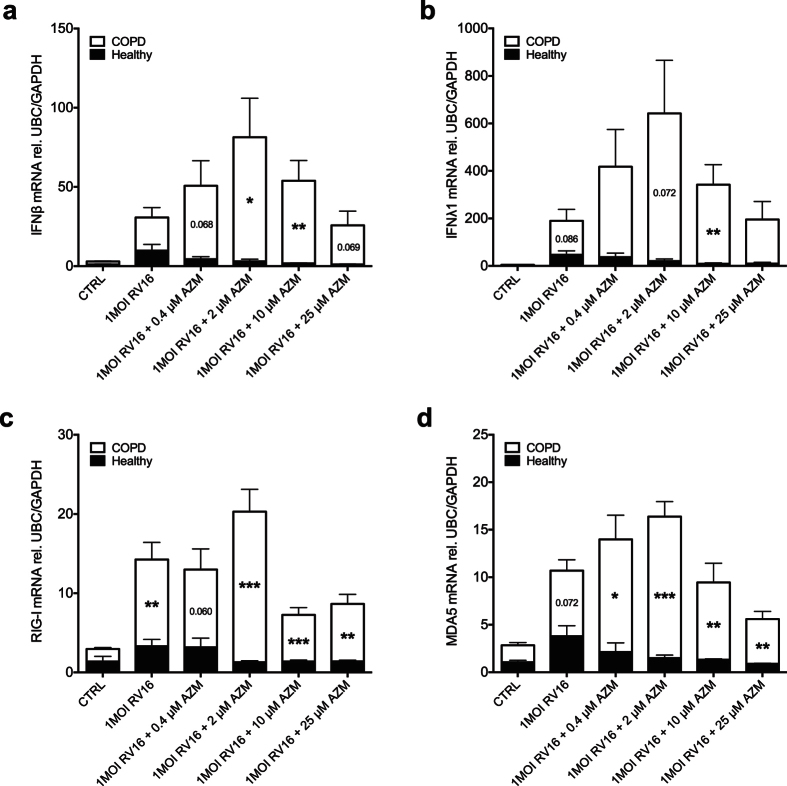
Azithromycin (AZM) augments rhinovirus-induced anti-viral pathways in COPD but not in healthy bronchial epithelial cells. HBECs from COPD patients and healthy subjects were pre-treated with azithromycin for 24 h before infection with 1MOI RV16 and continuous throughout the experiment. Cells were harvested for gene expression analysis 24 h post infection. Gene expression levels of IFNβ (**a**), IFNλ1 (**b**), RIG-I (**c**) and MDA5 (**d**) were measured by real-time PCR and data is presented as mean ± standard error of the mean (SEM) fold change of healthy control relative to UBC/GAPDH expression. Comparison of different groups was performed by ANOVA. *p < 0.05, **p < 0.01, ***p < 0.001 healthy vs. COPD. Data was obtained from 5 COPD donors and 4 healthy subjects.

Next we determined whether azithromycin augmented RV16-induced interferons also in bronchial epithelial cells from healthy individuals. At 24 h post infection azithromycin did not alter viral-induced interferon expression in healthy epithelium contrasting its marked effects in diseased epithelial cells (p < 0.01, Fig. 5a,b).

### Expression of RIG-I like helicases is induced by a combination of rhinovirus and azithromycin in bronchial epithelial cells from COPD donors but not in healthy cells

Infection with rhinovirus only modestly induced RIG-I and MDA5 expression, which was not altered by treatment with azithromycin at 24 h post infection in healthy epithelial cells. Thus, azithromycin enhanced viral-induced expression of RIG-I like helicases in epithelial cells from COPD patients to levels that were significantly higher than in healthy subjects (p < 0.001, Fig. 5c,d).

### Azithromycin reduces viral load

**Figure 6:**
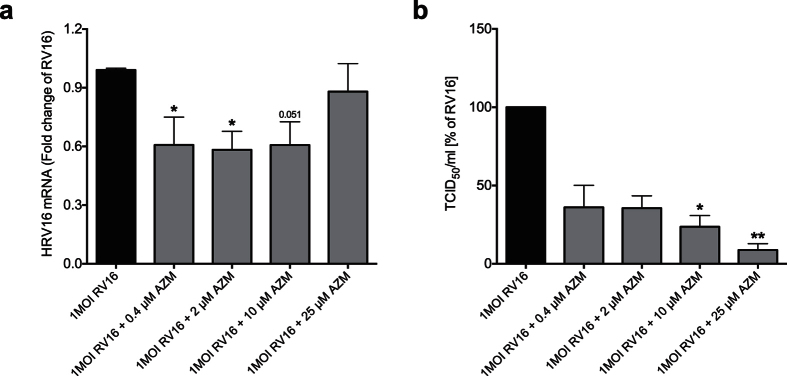
Azithromycin (AZM) reduces viral load in rhinovirus-infected bronchial epithelial cells. HBECs were pre-treated with azithromycin for 24 h before infection with 1MOI RV16 and continuous throughout the experiment. Cells were harvested for gene expression analysis 24 h post infection. Gene expression levels of viral load in COPD patients (**a**) were measured by real-time PCR and data is presented as mean ± standard error of the mean (SEM) fold change of RV16 relative to UBC/GAPDH expression. Additionally, viral progeny in cell supernatants was measured by TCID_50_ assay (**b**) and percent inhibition to RV16 was calculated. Comparison of different groups was performed by Kruskal-Wallis with Wilcoxon post testing. *p < 0.05, **p < 0.01 vs. RV16. Data was obtained from 5 COPD donors.

Next we investigated if the azithromycin-induced over-expression of endogenous interferons was associated with any reduction in viral load. As shown in Fig. 6 azithromycin treatment significantly reduced both viral load (Fig. 6a) and viral progeny in diseased epithelium 24 h post infection as measured by TCID_50_ assay (Fig. 6b).

### Budesonide does not reduce azithromycin-induced interferon expression

**Figure 7:**
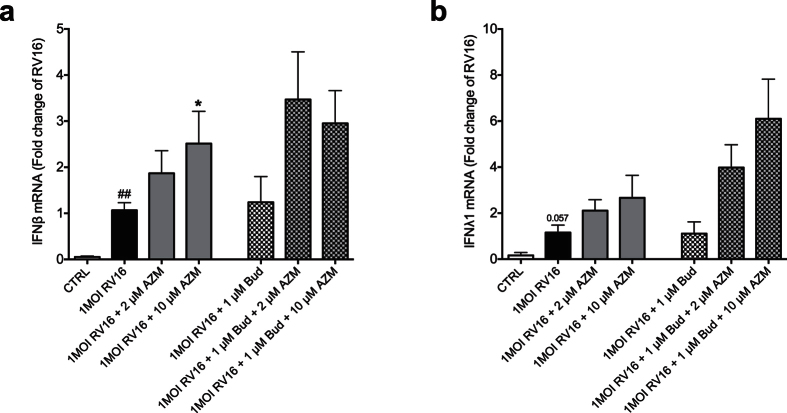
Addition of budesonide (Bud) does not reduce azithromycin’s (AZM) interferon inducing effect in rhinovirus-infected bronchial epithelial cells from COPD patients. HBECs from COPD patients were pre-treated with azithromycin for 24 h before infection with 1MOI RV16 and continuous throughout the experiment. Budesonide was added just after viral infection. Cells were harvested for gene expression analysis 24 h post infection. Gene expression levels of IFNβ (**a**) and IFNλ1 (**b**) were measured by real-time PCR and data is presented as mean ± standard error of the mean (SEM) fold change of RV16 relative to UBC/GAPDH expression. Comparison of different groups was performed by Wilcoxon. ^#^p < 0.05, ^##^p < 0.01 RV16 vs. control; *p < 0.05 AZM vs. RV16. Data was obtained from 5 donors.

A clinical trial involving daily azithromycin as a prophylactic therapy reduced exacerbations of COPD especially in patients requiring most intense drug treatment including corticosteroids^28^. We thus examined effects in our test system of a combination of budesonide, a corticosteroid, and azithromycin. Bronchial epithelial cells from COPD patients were treated with azithromycin 24 h prior to infection with RV16 and throughout the experiment. Budesonide was added immediately after RV16 infection. Budesonide alone did not alter viral-induced interferon expression but showed a slight tendency at enhancing azithromycin’s interferon-inducing effect 24 h post infection (Fig. 7).

## Discussion

This study demonstrates several novel aspects regarding anti-viral actions of azithromycin in primary epithelial cells obtained by bronchial brushings in COPD patients. First, it transiently and potently induced interferon expression already in un-infected cells. Second, when this response had subsidized, azithromycin still potently augmented rhinovirus infection-induced interferon expression in epithelial cells from COPD donors. Third, we did not observe significant interferon-inducing effects of azithromycin in rhinovirus-infected epithelial cells from healthy donors. The present findings are of special interest because the positive actions of azithromycin were obtained at clinically relevant concentrations and were associated with activation of molecular signalling pathways as well as functional effects on viral load.

Although small, the cohort of bronchial epithelial cell donors in this study revealed a marked distinction between health and disease. Thus, azithromycin caused increased expression of anti-viral interferons exclusively in COPD epithelium. The effects were generally concentration-dependent, in the range of 0.4–10 μM, strongly supporting the likelihood that these actions can be induced by treatment with clinical doses of azithromycin.

The importance of including several different concentrations was underscored by the observation that the concentration-response relationship exhibited a decline at concentrations above 10 μM. Such a clock-formed curve means that azithromycin’s effect can be missed if too few and high concentrations are used. Although the levels are supra-therapeutic for systemic use of azithromycin, the clock-form needs consideration. For example, it can prove a problem with topical use of this drug including any tentative future use of inhaled doses of azithromycin where sites of impact with the target epithelial cells may exhibit high drug concentrations.

The present findings apparently contrast observations by other groups reporting interferon-stimulant effects of 50 μM of azithromycin in bronchial epithelial cells from healthy donors^23^. The possibility that different cohorts of healthy individuals can respond differently to such high concentrations of azithromycin cannot be excluded. It is also possible that technical issues including the different chemicals used to dissolve azithromycin may have contributed. Of greater interest for future studies than studying effects of supra-therapeutic drug levels could be a follow-up on our observation that the doses of azithromycin employed in this study significantly reduced viral load and viral progeny in COPD epithelium. Can this effect be confirmed including our observation that it apparently occurred also without being associated with increased interferon expression?

Consistent with its effect on interferon expression, azithromycin concentration-dependently up-regulated RIG-I and MDA5 expression. Hence, azithromycin’s effect on interferons and RIG-I like helicases exhibited almost the same concentration-dependency and they occurred exclusively in bronchial epithelial cells from COPD donors. The association also was independent of presence or absence of rhinovirus infection. RIG-I helicases sense viral dsRNA with their helicase domain. This is believed to lead to conformational changes in which the CARD domain becomes accessible^29^ and triggers activation of downstream signalling via MAVS/VISA/Cardiff/IPS-1 and then TBK-1 leading to the activation of IRF3 and IRF7 that induce interferon expression^30^. We found a significant correlation between IFNβ expression and MDA5 expression for a concentration of azithromycin that was in the midst of the concentration response as regards the interferon-inducing effect. There was no such correlation with RIG-I. Differential involvement of these pattern recognition receptors in interferon stimulation by azithromycin needs further investigation. However, if induction of pattern recognition receptor expression by azithromycin actually is causing the increased expression of interferons can only be speculated at this stage. Furthermore our analysis of interferons, similarly to Gielen, *et al*.^23^ and Schogler, *et al*.^24^, focused on assessing gene expression levels, as levels of secreted interferons, measured by ELISA, were below detection limit, as also reported in other cohorts^31^.

Azithromycin induced IFNβ and IFNλ1 in epithelium from COPD donors in cells that had not been infected. This effect was further demonstrated as a marked up-regulation of interferon expression at an early point in time when infection alone had not yet produced more than marginal interferon effects. At a later time point when interferon-inducing effects of azithromycin apparently had ceased, this drug, again at low concentrations and dose-dependently, clearly augmented the viral infection-evoked interferon expression. These novel observations suggest that azithromycin, without additional viral stimulus, had caused a sustained priming effect making epithelial cells better equipped to combat bronchial viral infections. The present observations support the further evaluation of azithromycin as a prophylactic treatment in exacerbation-prone COPD patients. This approach would potentially complement a currently promising possibility of using inhaled doses of IFNβ aiming at reducing exacerbations of asthma and COPD^32^.

While some studies suggest an inhibitory effect of macrolides on CXCL8 expression^9^,^33^, others did not observe an effect^34^. However, these studies only used 14-membered macrolides and no viral stimulus. Azithromycin only modestly reduced viral-induced TNFα expression and did not alter viral-induced CXCL8 expression in the present epithelial cells from diseased donors, suggesting that azithromycin does not exhibit anti-inflammatory properties in this setting. Considering that interferon expression is mainly IRF3/IRF7-mediated while expression of TNFα and CXCL8 is mainly NFκB-mediated, it appears that azithromycin predominantly affects IRF signalling pathways after viral infection.

A major clinical study has now demonstrated efficacy of daily azithromycin treatment in preventing exacerbations in COPD^28^. Further analyses of the study data revealed that the prophylactic use of azithromycin produced greatest effect in patients already under heavy treatment with antibiotics and corticosteroids^28^. This observation made it of interest to examine effects on expression of interferons in diseased epithelial cells exposed to both azithromycin and a steroid such as budesonide used in treatment of COPD. We hypothesized that the present effects of azithromycin on interferon expression would not be inhibited by additional exposure of the epithelial cells to budesonide. Indeed, if anything the present observations suggested that budesonide tended to increase interferon-inducing actions of azithromycin. This is surprising, as budesonide is not known to stimulate interferon expression^35^. Our findings together with the clinical data by Han *et al*.^28^ suggest that a more detailed and versatile study of anti-viral potential of concomitant azithromycin and corticosteroid treatment would be of interest.

The present study demonstrates several novel features of azithromycin within a segment of its multiple pharmacological actions that has so far received limited attention. Hence, several of our findings are novel, leading to further studies rather than providing a definitive report on actions and involved mechanisms. In this exploratory study we used rather small cohorts of donors of epithelial cells. However, the clear-cut responses and differences recorded in this study indicate that the material was sufficient for revealing interferon-inducing effects of azithromycin in epithelium from COPD donors. Yet, further studies are warranted to explore the extent of differences between health and disease, including potential differences depending on severity of COPD. Our findings on association between effects on RIG-I like helicases need validation as regards causal involvement in the increased expression of interferons. Currently we also know too little regarding increased expression of epithelial interferon gene expression, protein production and release, and actual roles in combating viral infections. Perhaps the most urgent limitation of the present knowledge is the current lack of studies regarding *in vivo* effects of azithromycin on lung interferon expression and possible roles of this drug in protection against pulmonary viral infections.

## Conclusion

Possibly by inducing RIG-I like helicases clinically relevant concentrations of azithromycin concentration-dependently increased expression of type I and III interferons in COPD but not healthy bronchial epithelial cells. The stimulant effects of azithromycin were also transiently pronounced in un-infected COPD epithelium. Our data indicate that azithromycin-exposed COPD epithelium was primed to over-express the helicases and interferons when infected by rhinovirus. Azithromycin also reduced viral load. We suggest that azithromycin-induced epithelial interferon expression may contribute to the prophylactic effect of this drug in reducing exacerbations of COPD.

## Methods

### Chemicals

The macrolide antibiotic azithromycin was purchased from Sigma-Aldrich (Stockholm, Sweden) and dissolved in dimethyl sulfoxide (DMSO; Sigma-Aldrich) to a stock concentration of 10 mg/ml. The corticosteroid budesonide was purchased from Sigma-Aldrich (Stockholm, Sweden) and dissolved in DMSO to a stock concentration of 10 mM. Azithromycin and budesonide were further diluted in bronchial epithelial growth medium to a working concentration of 0.4 μM to 50 μM for azithromycin and 1 μM for budesonide. The final concentration of DMSO ranged from 0.003% to 0.37%. The highest concentration of DMSO was used as vehicle control as previously described^27^.

### Bronchial epithelial cell culture

Primary cell cultures of human bronchial epithelial cells (HBECs) were obtained from bronchial brushings of patients with COPD (characterized in Table 1). The study was approved by the regional ethical review board at Lund University (Ethical permit no. 218/2011) and was in accordance with ethical guidelines. Written informed consent was obtained from all study participants. Epithelial brushings were retrieved by bronchoscopy with a fibre optic bronchoscope (Olympus, IT160, Tokyo, Japan). For brushings standard sterile-sheared nylon cytology brushes were used to sample epithelial cells from bronchi. The procedure was performed in accordance with standard published guidelines and processed as previously described^36^. Additionally, human bronchial epithelial cells from 4 healthy donors (Lonza, Walkersville, MD, USA) were used.

Bronchial epithelial cells were cultured in bronchial epithelial growth medium (BEGM; Clonetics, San Diego, CA, USA). For experiments, HBECs were seeded into 12-well plates (Nunc Technologies, Carlsbad, CA, USA) and when 80–90% confluent (corresponding to ~100,000 cells/well) cells were exposed to azithromycin (in concentrations ranging from 0.4 to 50 μM) 24 h prior to infection with rhinovirus and continuous throughout the experiment. In a subset of experiments budesonide (in a concentration of 1 μM) was added to the cells after infection with rhinovirus.

### Infection with HRV16

For all experiments cells were used at passage 2–4. HBECs were infected with the major group rhinovirus RV16, grown in Ohio HeLa cells (European Collection of Cell Cultures) as described previously^37^ and RV16 was obtained from clarified cell lysates. HBECs were infected with RV16 at 1 multiplicity of infection (MOI; TCID_50_ 1.58 × 10^5^) for 1 h at room temperature while shaking. Then the virus was removed and cells were washed with phosphate buffered saline (PBS). Fresh BEGM medium containing azithromycin was added. Eight hours and 24 h post infection cells were lysed and harvested for gene expression analysis and 48 h post infection cells were lysed and harvested for protein expression analysis.

### RNA extraction and quantification of gene expression by RT-qPCR

Total RNA was extracted from HBECs using a RNA extraction kit (Nucleospin^®^ RNA II, Macherey-Nagel, Düren, Germany). Then 1 μg of RNA was reverse transcribed to cDNA (Precision Nanoscript Reverse Transcription Kit, PrimerDesign, Southampton, UK) and real-time quantitative PCR was performed. In brief, a Mx3005P qPCR system (Stratagene, La Jolla, CA, USA) with standard cycling parameters was used to perform thermo-cycling and real-time detection of PCR products. The following primer sequences (PrimerDesign, Southampton, UK) were used:

IFNβ: TTACTTCATTAACAGACTTACAGGT (forward) and TACATAGCCATCGTCACTTAAAC (reverse),

IFNλ1: ATGGGAACCTGTGTCTGAGAA (forward) and GGGTGAGAGGAAATAAATTAAGGAA (reverse),

RIG-I: TTCTCTTGATGCGTCAGTGATA (forward) and CCGTGATTCCACTTTCCTGAA (reverse),

MDA5: CCGTGATTCCACTTTCCTGAA (forward) and TTATACATCATCTTCTCTCGGAAATC (reverse),

HRV16: GAGAGGTTAAGAACTTGATTGAA (forward) and CTAATTTTGTTTGTGGTGATAGAG (reverse),

CXCL8: CAGAGACAGCAGAGCACAC (forward) and AGCTTGGAAGTCATGTTTACAC (reverse),

TNFα: AGGTTCTCTTCCTCTCACATAC (forward) and ATCATGCTTTCAGTGCTCATG (reverse).

Samples were analysed by the ΔΔCt method^38^ and related to UBC/GAPDH expression. Groups were normalized to untreated control or RV16 when appropriate.

### Western Blot analysis of RIG-I like helicases

Protein expression of RIG-I and MDA5 was quantified by western blot. Experiments were performed as described and cells were lysed 48 h post RV16 infection using a lysis buffer containing 1% TritonX-100, 10 mM Tris-HCl, 50 mM NaCl, 5 mM EDTA, 30 mM Na_4_P_2_O_7_, 50 mM NaF, 0.1 mM Na_3_VO_4_, 1% phosphatase and protease inhibitors (Sigma-Aldrich, Stockholm, Sweden). Protein concentrations were determined by BCA protein assay (Pierce Thermo Scientific, Waltham, MA, USA) for each sample and equal amounts of protein were loaded and electrophoresed on a 10% TGX stain-free gel (Bio-Rad Laboratories AB, Solna, Sweden), before blotting on a Trans-Blot Turbo Transfer System (Bio-Rad Laboratories AB, Solna, Sweden). This was followed by blocking of the membrane in 5% (w/v) milk in Tris-buffered saline Tween-20 and overnight incubation at 4 °C with primary antibodies (anti RIG-I Rabbit mAb, anti MDA5 Rabbit mAb, anti GAPDH Rabbit mAb; Cell Signaling Technology, Leiden, The Netherlands). Then the membrane was washed and incubated for 1 h with secondary antibodies (anti Rabbit IgG HRP-linked Ab; Cell signalling Technology, Leiden, The Netherlands). Detection was performed by chemiluminescence using SuperSignal West FEMTO substrate (Pierce Thermo Scientific, Waltham, MA, USA) and immunoblots were visualized by LI-COR Odyssey Fc Imager (LI-COR Biosciences, Lincoln, NE, USA) and Image Studio (v.3.1.4; LI-COR Sciences, Lincoln, NE, USA).

### TCID_50_ assay for viral progeny

Supernatants from RV16-infected HBECs were collected 24 h post infection and 1:2 serial diluted in DMEM with Glutamax containing 2% FBS, 1% penicillin-streptomycin, 1% non-essential amino acids and 1% sodium pyruvate (Life Technologies, Carlsbad, CA, USA). Dilutions were added to 80% confluent Ohio HeLa cells (European Collection of Cell Cultures) in 96-well plates (Nunc Technologies, Carlsbad, CA, USA) in duplicates. Plates were rocked for 1 h at room temperature and then incubated for 4 days at 37 °C, 5% CO_2_. Then the cell monolayer was fixed and stained with crystal violet. Cytopathic effects were assessed by spectrophotometry and TCID_50_ was calculated with the Spearman-Kärber algorithm.

### Cell viability

Cell morphology of HBECs was regularly observed by light microscopy at different time points after treatment with azithromycin and/or budesonide and after infection with RV16. Furthermore LDH levels were measured in cell supernatants according to the manufacturer’s instructions (Roche Diagnostics, Bromma, Sweden). These data confirmed that neither azithromycin nor budesonide did induce cell death in epithelial cells.

### Statistical analysis

Data is presented as mean with standard error of the mean. Statistical analysis was performed in R^39^. P-values < 0.05 were regarded statistically significant. Experiments were repeated up to 3 times for each donor.

## Additional Information

**How to cite this article**: Menzel, M. *et al*. Azithromycin induces anti-viral effects in cultured bronchial epithelial cells from COPD patients. *Sci. Rep.* **6**, 28698; doi: 10.1038/srep28698 (2016).

## Supplementary Material

Supplementary Information

## Figures and Tables

**Table 1:** Patient characteristics of the COPD donors included in this study.

Gender	Age [years]	Pack-years	FEV_**1**_ **[pred%]**	GOLD stage	Concomitant medication
Male	69	100	53.9	GOLD 2	LAMA, LABA, ICS
Male	70	46	52.2	GOLD 2	LAMA, LABA, ICS
Male	77	20	47.0	GOLD 3	LAMA, LABA, ICS
Female	64	60	52.0	GOLD 2	ICS, LABA
Female	62	50	50.0	GOLD 2	ICS, LABA
Female	62	50	52.0	GOLD 2	ICS, LABA
Male	61	40	77.0	GOLD 2	LABA

Pack-years, packs of cigarettes smoked per day multiplied by the number of years smoked; FEV_1_, forced expiratory volume in 1 s; GOLD staging based on degree of airflow limitation; LAMA, long-acting muscarinic antagonist; LABA, long-acting beta-adrenoceptor agonist; ICS, inhaled corticosteroids.
