# Supplementary material for: Azithromycin induces anti-viral effects in cultured bronchial epithelial cells from COPD patients
Source: Sci Rep. 2016 Jun 28;6:28698. doi: 10.1038/srep28698 (PMC4923851; doi:10.1038/srep28698)
Supplement: Supplementary Information [file srep28698-s1.pdf]

Azithromycin induces anti-viral effects in cultured bronchial epithelial cells from COPD patients

Mandy Menzel[1], Hamid Akbarshahi[1], Leif Bjermer[2], Lena Uller[1]*

Affiliation:

[1] Respiratory Immunopharmacology, Department of Experimental Medical Science, Lund University, Sweden.

[2] Lung medicine and Allergology, Department of Clinical Sciences, Lund University, Sweden.

* Corresponding author:

Lena Uller, Department of Experimental Medical Science, Lund University, BMC D12, 22184 Lund, Sweden. E-mail: Lena.Uller@med.lu.se.

Supplementary figure S1: Azithromycin (AZM) does not display cytotoxic effects in primary bronchial epithelial cells from COPD patients. HBECs from COPD patients were pre-treated with azithromycin for 24h before infection with 1MOI RV16 and continuous throughout the experiment. Alternatively, HBECs from COPD patients were pre-treated with azithromycin for 24h and continuous throughout the experiment. Cell supernatants were analysed for LDH levels. Comparison of different groups was performed by Kruskal-Wallis with Wilcoxon post testing. #: $p < 0.05$, ##: $p < 0.01$ RV16 vs. control; *: $p < 0.05$ AZM vs. RV16. Data was obtained from 4 donors.

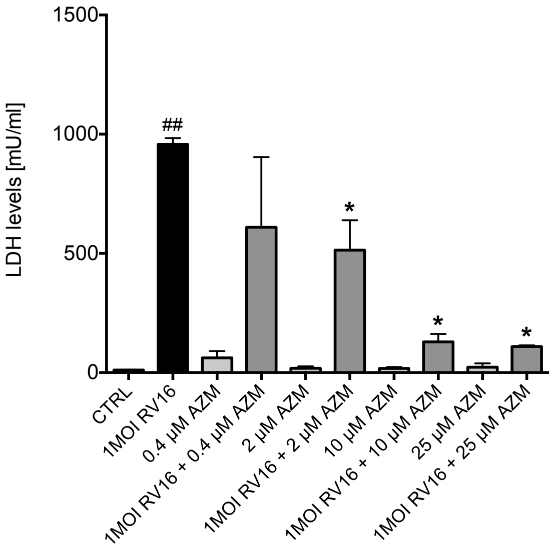
LDH levels [mU/ml]
1500
1000
500
0
##
*
*
*
CTRL
1MOI RV16
0.4 µM AZM
1MOI RV16 + 0.4 µM AZM
2 µM AZM
1MOI RV16 + 2 µM AZM
10 µM AZM
1MOI RV16 + 10 µM AZM
25 µM AZM
1MOI RV16 + 25 µM AZM

Supplementary figure S2: Azithromycin (AZM) induces early expression of RIG-I like helicases independent of rhinoviral infection in bronchial epithelial cells from COPD patients. HBECs from COPD patients were pre-treated with azithromycin for 24h before infections with 1MOI RV16 and continuous throughout the experiment. Cells were harvested for gene expression analysis 8h post infection (a, b). HBECs from COPD patients were pre-treated with azithromycin for 24h and continuous throughout the experiment. Cells were harvested for gene expression analysis at 8h (c, d). Gene expression levels of RIG-I (a, c) and MDA5 (b, d) were measured by real-time PCR and data is presented as mean ± standard error of the mean (SEM) fold change of control relative to UBC/GAPDH expression. Comparison of different groups was performed by Kruskal-Wallis with Wilcoxon post testing or Dunn's post testing. #: $p < 0.05$ RV16 vs. control; *: $p < 0.05$, **: $p < 0.01$ vs. RV16. Data was obtained from 5 donors. A representative Western Blot image of RIG-I (e) and MDA5 (f) protein is shown.

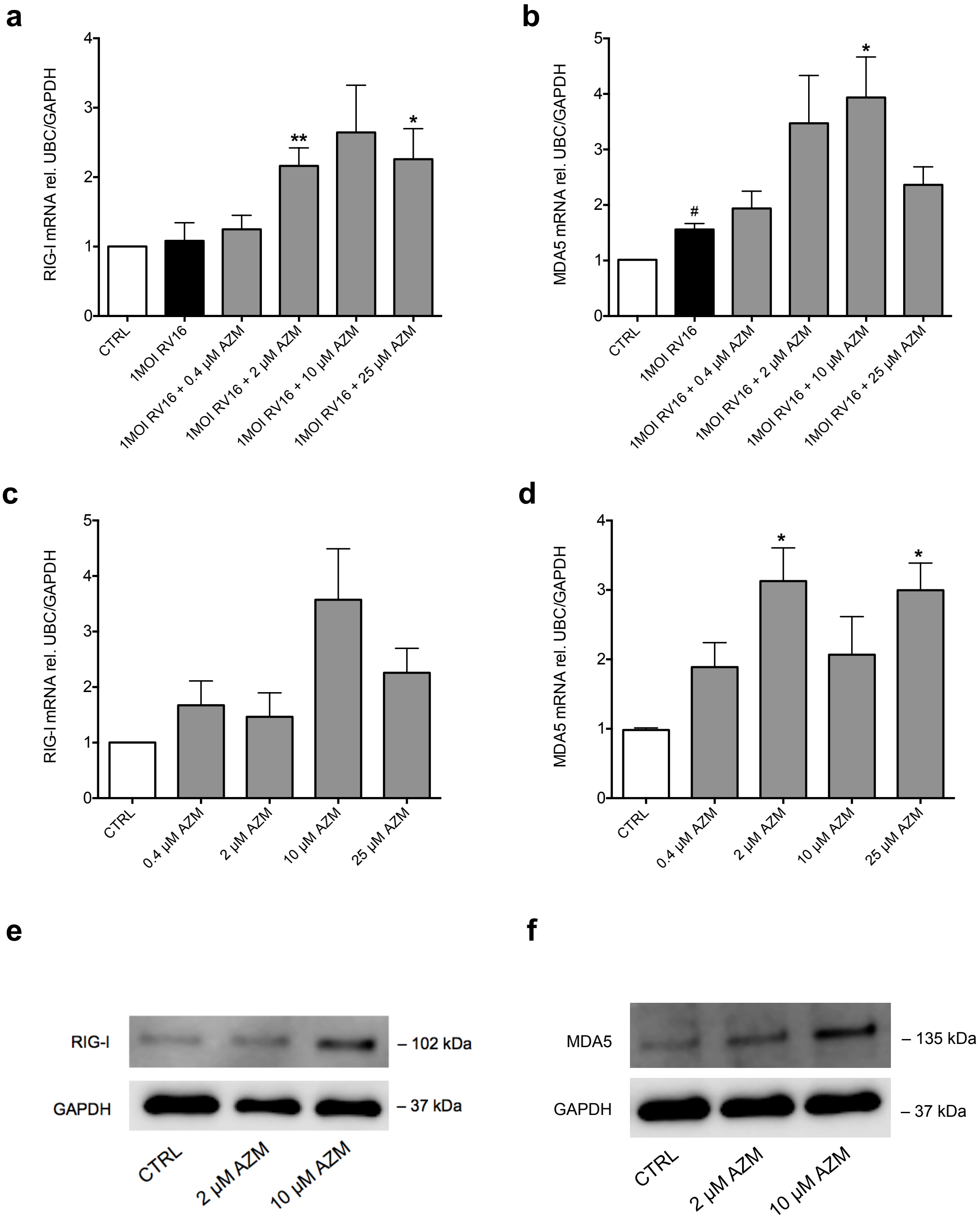
a
RIG-I mRNA rel. UBC/GAPDH
CTRL
1MOI RV16
1MOI RV16 + 0.4 µM AZM
1MOI RV16 + 2 µM AZM
1MOI RV16 + 10 µM AZM
1MOI RV16 + 25 µM AZM
b
MDA5 mRNA rel. UBC/GAPDH
c
RIG-I mRNA rel. UBC/GAPDH
0.4 µM AZM
2 µM AZM
10 µM AZM
25 µM AZM
d
MDA5 mRNA rel. UBC/GAPDH
e
RIG-I
GAPDH
– 102 kDa
– 37 kDa
f
MDA5
– 135 kDa

Supplementary figure S3: Azithromycin (AZM) treatment alone has no interferon-inducing effect at 24h in bronchial epithelial cells from COPD patients. HBECs from COPD patients were pre-treated with azithromycin for 24h and continuous throughout the experiment. Cells were harvested for gene expression analysis at 24h. Gene expression levels of IFNβ (a) and IFNλ1 (b) were measured by real-time PCR and data is presented as mean ± standard error of the mean (SEM) fold change of control relative to UBC/GAPDH expression. Comparison of different groups was performed by Kruskal-Wallis with Dunn's post testing. Data was obtained from 3 donors.

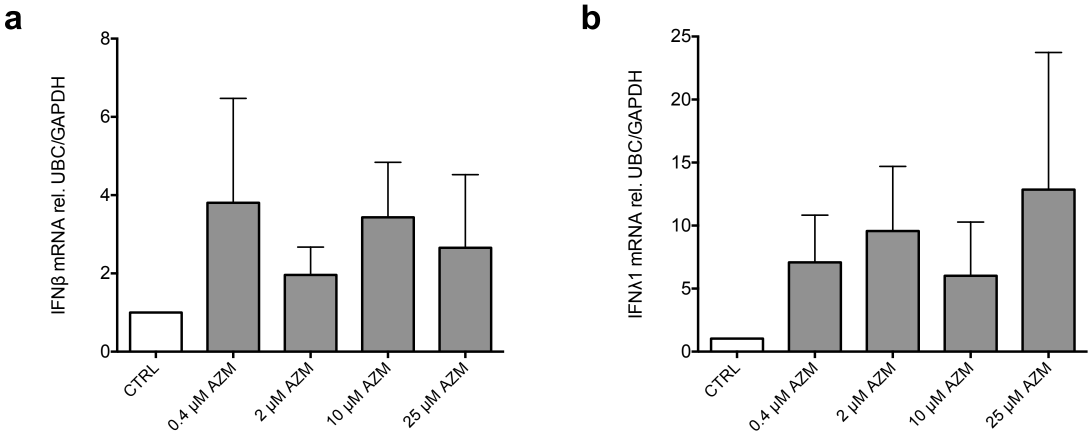
a
b
IFNβ mRNA rel. UBC/GAPDH
IFNλ1 mRNA rel. UBC/GAPDH
0
2
4
6
8
5
10
15
20
25
CTRL
0.4 µM AZM
2 µM AZM
10 µM AZM
25 µM AZM
